# An Extremely Rare Cause of Isolated Congenital Anosmia

**DOI:** 10.1155/2022/9692716

**Published:** 2022-07-07

**Authors:** Chia Saw, Noel David Friesen, Anthony Bartley

**Affiliations:** ^1^Consultant Paediatrician, SJOG Midland Hospital, Midland, Australia; ^2^Consultant Paediatric Radiologist, Imaging Department of Perth Children's Hospital, Perth Radiological Clinic, Perth, Australia

## Abstract

A 14-year-old adolescent was referred to a regional paediatric outpatient clinic with anosmia by her family doctor in Western Australia. The patient has no recollection of her previous ability to smell, suggesting the possibility of congenital anosmia. She was assessed in the paediatric outpatient clinic. A “noncontrast high-resolution MRI-brain scan with Anosmia-Protocol” was requested as the first-line investigation of choice by the treating paediatrician. The MRI was reported *as* “absence of olfactory tracts with preserved olfactory bulb volume.” We report an extremely rare case of “isolated agenesis of the olfactory tract with intact olfactory bulbs” and discuss the clinical approach in bedside assessment of isolated congenital anosmia (ICA). Congenital anosmia can be a presentation of olfactory bulb aplasia; however, little is known about isolated olfactory tract agenesis and its treatment options. The patient was counselled on the diagnosis and safety advice provided.

## 1. Background and Aim

Olfactory dysfunction is not uncommon in the general population and its prevalence increases with age [1]. Around 5% of individuals over the age of 45 years old have olfactory dysfunction in the form of hyposmia or anosmia. Most of them are caused by degenerative or acquired conditions [2]. Congenital anosmia (CA), however, has a much lower prevalence, affecting 1 in 10,000 individuals, and the majority of them present late in life with no recollection of their ability to smell [2]. Congenital anosmia in the paediatric population is commonly associated with a syndrome or genetic disorder. It can also be an acquired condition secondary to craniofacial trauma, endocrine disorders, or brain tumours. Isolated congenital anosmia (ICA) is usually caused by aplasia or hypoplasia of the olfactory bulb [3, 4].

ICA is an extremely rare condition [5]. It is often diagnosed late as it is not associated with any endocrine or genetic disorder. One study showed that the diagnosis of ICA can be delayed for up to 13 years from the time of reported concerns due to its subtle nature of presentation with lack of other associated features [5]. Acquired olfactory dysfunction, on the other hand, is often diagnosed and managed early.

We discuss a relatively straightforward clinical assessment and diagnostic approach in establishing the cause of ICA in a regional paediatric outpatient setting in Western Australia.

## 2. Case Report

An adolescent was referred by her general practitioner to the paediatric outpatient specialist clinic for “inability to smell” since a “young age.” The patient has no recollection of any previous sense of smell. She has no previous medical illnesses. No other family members were reported to have anosmia or hyposmia.

Physical examination revealed a postpubertal and a nondysmorphic child. Her upper nasal passage was noted to be patent without any hypertrophied nasal turbinate or obstruction. The neurological and cardiorespiratory examinations were unremarkable. In this regional hospital, subspecialty support like ear nose throat (ENT) and neurology consultation was not readily available. A quick informal bedside “wafting smell test” was carried out using some readily available bedside substances like coffee beans and alcohol hand gel by the duty paediatrician. Based on the history and physical examination, an impression of “isolated anosmia with a likely congenital etiology” was made. MRI of the brain with anosmia protocol was ordered as the first-line investigation of choice, which revealed a rare case of olfactory tract agenesis with an intact olfactory bulb (as discussed below).

## 3. Interpretation

Following an initial clinical assessment of the index patient, there was a strong indication to suggest that the patient's anosmia was congenital in nature. An “MRI Brain with Anosmia Protocol” was ordered as the investigation of choice which revealed the diagnosis as discussed below.

**Figure 1:**
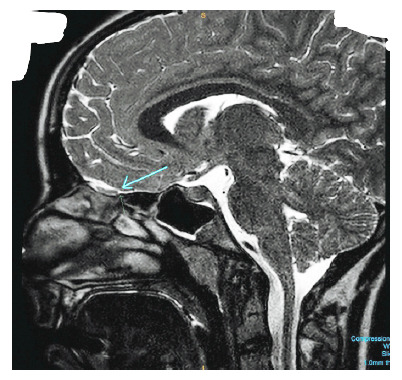
T2-weighted high-resolution image of the MRI brain anosmia protocol sagittal view showing normal volume olfactory bulb with sudden termination and discontinuation, suggesting olfactory tract agenesis (blue arrow).

**Figure 2:**
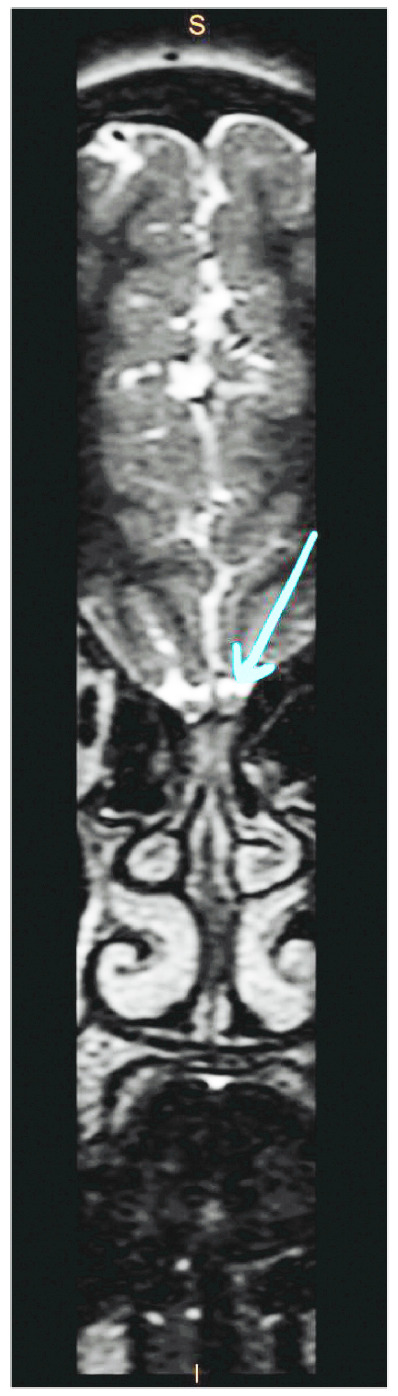
Coronal view of the MRI brain with olfactory protocol showing normal volume of olfactory bulbs (blue arrow).

**Figure 3:**
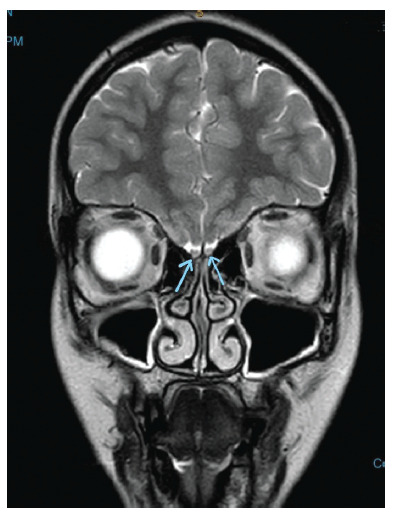
Coronal view showing the presence of the olfactory bulb bilaterally (blue arrow).

**Figure 4:**
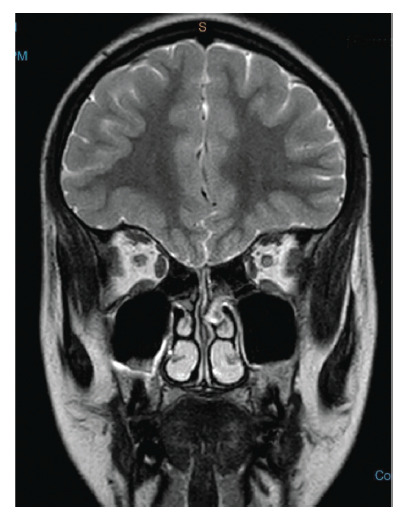
Coronal view showing the absent olfactory tract.

**Figure 5:**
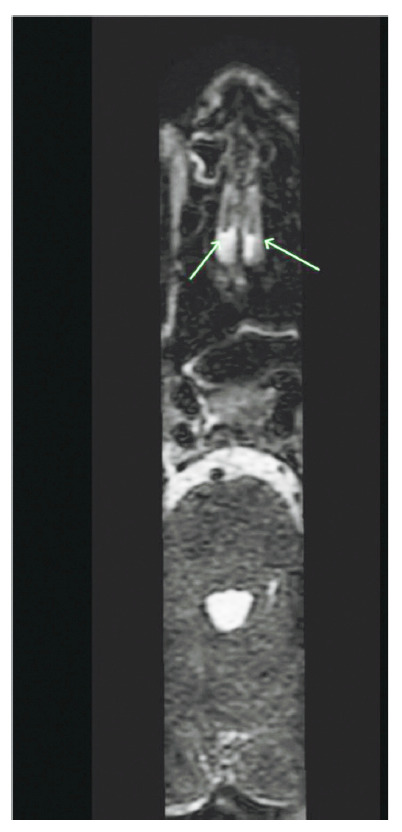
Transverse view of MRI anosmia protocol illustrating the presence of a normal volume olfactory bulb but absent olfactory tracts.

Due to the lack of clinical suspicion for other associated genetic or endocrine disorders in this index patient, there was no indication for other imaging and blood investigations. Further genetic testing was deemed unnecessary as there was no reported family history of anosmia (Figures 1–5)

The MRI technique adopted was a “Noncontrast Anosmia Protocol” study performed with high-resolution T2 sequence through the anterior cranial fossa.

The formal reported findings from the MRI brain with anosmia protocol are as follows.

This is the report by the radiologist- to maintain originality, “I will not be altering the wording of the report of the MRI images.” There are normal appearances of the midline structures including the pituitary. The white matter returns a normal signal with no evidence of demyelination. The olfactory bulbs are present and of normal volume. However, these appear to end abruptly with the absence of the olfactory tracts. There are otherwise normal appearances of the anterior cranial fossa. The olfactory sulci are normally formed.

### 3.1. Final Conclusion on MRI Brain

1. The olfactory bulbs are present and of normal volume. However, there appears to be absence of the olfactory tracts, with the bulbs terminating abruptly. This is most likely congenital rather than related to prior trauma (of which there is no other evidence). No other abnormality is identified of the anterior cranial fossa and the midline structures are normal in appearance.
2. No intracranial abnormality is identified with no mass, infarction, or evidence of demyelination.
3. There is minimal mucosal thickening within the bilateral maxillary and left sphenoid sinuses. The paranasal sinuses are otherwise clear.

The olfactory system is one of the five main sensory systems in the human body. A functioning olfactory system requires normal anatomy of the nasal cavity (analogue to the conductive pathway of the auditory sensory system) coupled with a normal sensory neural pathway (olfactory bulbs and tracts).

The olfactory system is one of the most precocious sensory systems to develop in human embryology [6]. Its embryological pathway is divided into two major components: the olfactory epithelia (OE) and the olfactory bulb (OB) pathway [6]. Defects in any of these pathways would lead to olfactory dysfunction presenting as hyposmia or total anosmia.

Olfactory dysfunction is an uncommon presenting complaint in this regional paediatric outpatient setting. Most paediatric patients with olfactory dysfunction present with hyposmia rather than complete anosmia due to other acquired causes like craniofacial trauma, infection, adenoid hypertrophy, and malignancy [4]. Because of its subtle nature, olfactory dysfunction in the paediatric population is often underdiagnosed and underrecognised.

There are very limited case reports on isolated congenital anosmia [7]. One case report by Vowles et al. highlighted a few interesting presentations of its index patient [7]. The reported five-year-old girl was described to have drunk rancid milk without noticing. She was also not noticing the smell of smoke, which may lead to potentially life-threatening consequences during an emergency [7].

The causes of congenital anosmia in the paediatric population can be generally divided into those with underlying congenital anatomical defects or those associated with genetic or endocrine disorders (as discussed below), for example, congenital choanal atresia, olfactory bulb/nerve agenesis, CHARGE syndrome (OMIM #214800), Bardet- Biedl syndrome (OMIM #209900), Refsum disease (OMIM #266500), and Kallman syndrome (OMIM #308700).

Intriguingly, there is a recently published research revealing a series of people with normal olfactory function despite having a complete absence of the olfactory bulbs [8]. In this paper by Weiss et al., it was revealed that 0.6% of adult women in the general population sample were found to have a complete absence of the olfactory bulbs, but yet they have a normal functioning olfactory system [8].

**Figure 6:**
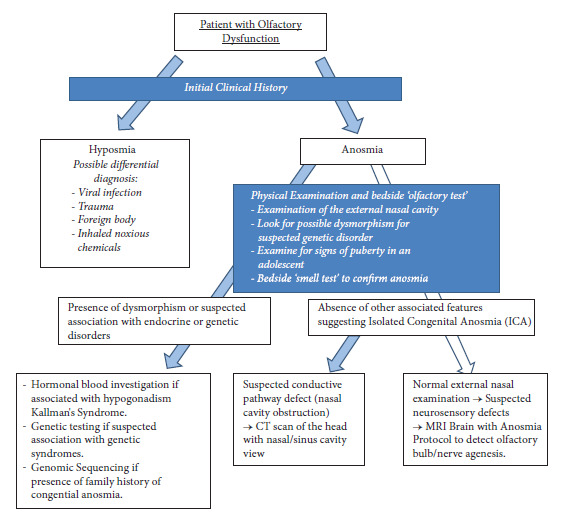
Suggested workflow for paediatric patient with olfactory dysfunction.

We further discuss the clinical approach to a child presenting with congenital anosmia in a regional paediatric outpatient setting (without other subspecialty services) as illustrated below (Figure 6).

## 4. Treatment Options

Congenital isolated anosmia (ICA) caused by aplastic olfactory bulb/tract has limited treatment options known to date. Counselling and safety advice form the pillar of the management plan for such patients. No further active medical or surgical intervention was thought to be available for this index patient following a discussion with the paediatric neurologist and ENT team. General safety advice was provided to ensure the patient would be able to avoid potential life-threatening conditions like having a functioning smoke and gas detector at home, avoid using a gas cooker, and to be more cautious on reading food expiry date labels prior to consumption.

## Data Availability

No data were used to support this study.

## Additional Points

*Learning Points.* Isolated congenital anosmia in the paediatric population is an extremely rare condition. It is often diagnosed late due to its nature of presentation. There is no treatment option known to date for congenital aplasia/agenesis of the olfactory bulb and/or olfactory tract. Safety advice forms the pillar of management for such patients as anosmia can pose potential life-threatening consequences. Utilising easily available bedside substances for “smell tests” provided a crucial clinical impression in establishing the etiology of the diagnosis.
